# Genetic Differentiation of the Two Types of Polish Cold-blooded Horses Included in the National Conservation Program

**DOI:** 10.3390/ani10030542

**Published:** 2020-03-24

**Authors:** Artur Gurgul, Igor Jasielczuk, Ewelina Semik-Gurgul, Klaudia Pawlina-Tyszko, Tomasz Szmatoła, Grażyna Polak, Monika Bugno-Poniewierska

**Affiliations:** 1 Center for Experimental and Innovative Medicine, University of Agriculture in Kraków, Rędzina 1c, 30-248 Kraków, Poland; igor.jasielczuk@urk.edu.pl (I.J.); tomasz.szmatola@urk.edu.pl (T.S.); 2 Department of Animal Molecular Biology, National Research Institute of Animal Production, Krakowska 1, 32-083 Balice, Poland; ewelina.semik@izoo.krakow.pl (E.S.-G.); klaudia.pawlina@izoo.krakow.pl (K.P.-T.); 3 Department of Horse Breeding, National Research Institute of Animal Production, Krakowska 1, 32-083 Balice, Poland; grazyna.polak@izoo.krakow.pl; 4 Department of Animal Reproduction, Anatomy and Genomics, University of Agriculture in Kraków, al. Mickiewicza 24/28, 30-059 Kraków, Poland; monika.bugno-poniewierska@urk.edu.pl

**Keywords:** draft horses, genetic differentiation, selection signatures, Sokólski, Sztumski

## Abstract

**Simple Summary:**

In this study we investigated the degree of genetic differentiation among two types of Polish cold-blooded horses, namely Sokólski and Sztumski. Both these horse types are a subject of conservation breeding as their populations have been dramatically reduced during recent decades. These cold-blooded horses are considered as separate breeds, with separate stud books, but yet still their genetic differentiation has not been precisely determined, especially on the genome–wide level. In this study, we characterized genetic differentiation of these two horse populations as well as their genetic variation, admixture patterns, and level of relatedness within populations with the use of single nucleotide polymorphism (SNP) genotyping arrays. We also attempted to detect genome regions with the largest genetic differences between those horses by the detection of so-called diversifying selection signals. The results of this study provide initial evidence supporting decisions made during conservation breeding program design and answer some of the questions raised by the breeders of Sokólski and Sztumski horses.

**Abstract:**

The current role of the horse as a companion animal resulted in a decrease of interest in breeding and usage of draft horses. This meant that the population of cold-blooded horses in Poland has been dramatically reduced during the last decades. To avoid impoverishment of the gene pool of the local horse population, a conservation program was established which involves draft horses and other local horse breeds. The draft horses bred in Poland can be subdivided in a few horse types of which the most widespread and consolidated are Sztumski and Sokólski horses. These two subpopulations are phenotypically diversified, however, the overall level of their genetic differentiation seems to be relatively low and not precisely determined, especially with the use of molecular markers. In reference to this, in this study we used Illumina genotyping arrays to describe in detail the genetic differentiation of these two cold-blooded horse populations. We describe the genetic distance between them, as well as within-population variation, admixture patterns, and level of relatedness within populations. We also made an attempt to detect genome regions divergently selected between those horses by the detection of diversifying selection signals. The results of this study provide initial evidence supporting breeding decisions that were made during conservation breeding program design and answer questions raised by the breeders of Sokólski and Sztumski horses concerning the level of their genetic variation and differentiation.

## 1. Introduction

The current population of draft horses in Poland was dramatically reduced due to the limited interest in usage and breeding of working horses. To maintain biodiversity and protect the existing gene pool, local draft horses were included in a conservation program which follows the guidelines of the FAO (Food and Agriculture Organization of the United Nations) Global Plan of Action for Animal Genetic Resources (Interlaken, Switzerland, 2007). Currently, two subpopulations (types) of a draft horse with separate stud books are maintained in Poland within the conservation program, namely Sokólski and Sztumski [1]. These populations are considered as separate breeds because of a clear heritable difference in their appearance, slightly different breeding history, and region of origin [2]. Sokólski horses were shaped by the influence of the environmental conditions of north-eastern Poland, which are characterized by a harsh climate and poorer quality of soils in relation to other regions of the country. The initial genetic material of these horses came from primitive local populations. These primitive local horses were a relatively small size but were characterized with high strength and resistance to cold and poor living conditions. Because of their light weight, however, primitive horses could not be used for transportation or heavier field work which prompted the search for horses of a larger mass and more generous cold-blooded type. Today’s Sokólski horse derives from crossbreeding of local Polish mares with imported Ardennes and Breton sires. Consistent and planned breeding work as well as rational feeding allowed the development of a specific cold-blooded population, characterized by a considerably thicker fat cover, well-defined tendons, mild temperament, endurance, and suitability to perform various sledging or riding tasks [1,2]. 

The other studied subpopulation of Polish cold-blooded horses is formed by the Sztumski horses which are the largest and the heaviest of the cold-blooded horses maintained in Poland. Sztumski horses were originally created on the basis of a local population crossbred mainly with Ardennes and Belgian sires. They were bred predominantly in the areas of Powiśle, Warmia, and Mazury, where heavy soil that is difficult to cultivate required particularly strong working horses. Thus, the Sztumski horses are characterized by a larger caliber and thicker fat cover in comparison to the Sokólski horses [1,2].

Despite these clear phenotypical differences between the Sokólski and Sztumski horses, several concerns have arisen regarding their genetic differentiation, mainly because of some stallion exchange events and past breeding history that was not fully registered in the breeding records. The existing pedigree analysis of 2653 Sztumski and Sokólski horses involved in the conservation programs in 2014 (12,821 ancestors) showed that the contribution of the Ardennes sires amounted to 61.9% for Sokólski and 64.2% for Sztumski horses, while Belgian sires amounted to 9.3% for Sokólski and 18.3% for Sztumski. The contribution of Breton founders in Sokólski populations was equal to 4.38%. The average inbreeding coefficients were 1.56% and 1.54%, whereas effective population size (Ne) was 493 and 482, in these populations, respectively. The average pedigree completeness for five generations was 98.24 for Sokólski horses and 98.79 for Sztumski [1]. To analyze in detail the current genetic differentiation of these two horse populations and to make an attempt to answer if their genetic characteristics differ in a manner that allows them to be considered as separate breeds and to determine if further maintaining of the separate stud book is justified, we performed whole genome scanning with the use of genotyping arrays to reveal global and regional genome differentiation of these populations. We used genetic differentiation measures to detect diversifying selection signatures among these horse types as well as applying genetics methods of other population to capture the signs of their genetic differentiation. Both these populations were included in our previous study concerning diversifying selection signatures among selected Polish horse breeds [3], however, their direct genetic differentiation was not analyzed as only measures of genetic distance between separate breeds or major horse types against all other studies breed were the subject of that investigation.

## 2. Materials and Methods 

**Figure 1:**
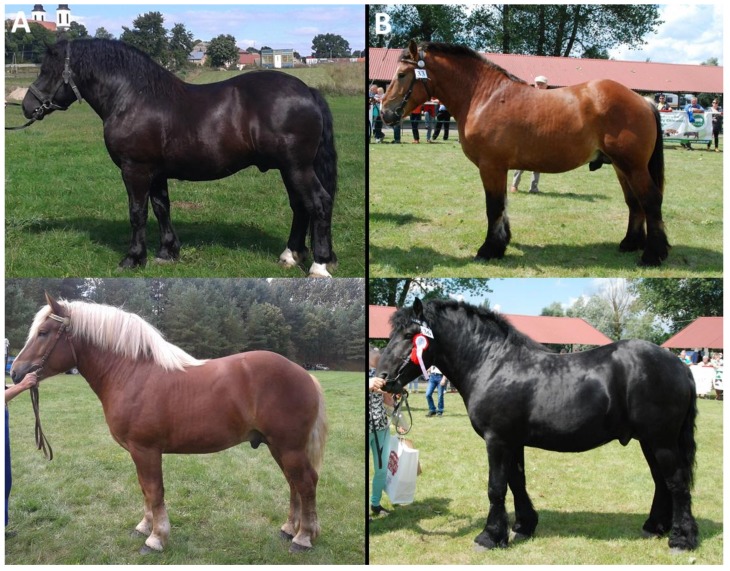
Three years old stallions of (**A**) Sokólski and (**B**) Sztumski horses (picture by G. Polak).

The study material was obtained from Sokólski (*n* = 107; SOK) and Sztumski (*n* = 69; SZTUM) horses (Figure 1) randomly sampled from breeding population during annual breeding classification so that they originated from different studs and different regions. Relatedness structure was analyzed by the evaluation of kinship coefficients derived from the genetic relationship matrix (GRM) calculated using Plink v1.90b4 software (-make-grm-gz command) [4]. Ten mL blood samples were collected from the jugular vein by a veterinary doctor to vials containing EDTA K3. The material was collected in herds participating in the programs for the conservation of genetic resources of Sztumski and Sokólski horses located in the Podlasie (Sokólski) and Pomorskie (Sztumski) provinces. Each breeder participating in the program signed a cooperation agreement with the National Research Institute of Animal Production (NRIAP) and undertook to provide data and biological material for research purposes. Breeders participating in the conservation program are obliged to keep animals in accordance with the criteria of animal welfare, which is subject to the control of district veterinarians. The animal procedures were approved by the Local Animal Care Ethics Committee No. II in Kraków (permission number 1293/2016), in accordance with EU regulations. Genomic DNA was purified from the blood samples using a Sherlock AX (A&A Biotechnology, Gdynia, Poland) kit and, after a quality control, was genotyped with the use of the Neogen Equine Community BeadChip assay (GGP Equine70k, Illumina, San Diego, CA, USA) including probes for 65,157 single nucleotide polymorphisms (SNPs), according to the standard Infinium Ultra protocol. 

SNP filtering was performed as previously described [3]. Only genotypes with call rate >0.97 were used for the analysis. The initial SNP set was filtered to remove markers located on the sex chromosomes and was further reduced by applying population-wide filters. The filters included MAF (minor allele frequency) threshold of 5% and <20% of missing genotypes in the whole studied population. Additionally, SNPs with critical *p*-value for Hardy-Weinberg equilibrium (HWE) < 1.0E-06 in each breed separately were excluded. The final SNP panel of 52,023 markers was scattered across the horse genome (EquCab2.0) with an average inter-marker distance of 43.0 kb.

The horse types’ genetic differentiation was evaluated using pairwise F_ST_ distances [5] measuring locus-specific allele frequency variation between populations. To detect diversifying selection signatures, SNP-by-SNP F_ST_ values obtained using Plink software v1.90b4 (--fst case-control command) [4] were averaged in 10-SNP sliding windows to account for random variation in locus-by-locus allele frequency. The window-averaged F_ST_ values were subsequently ranked and the top 1% of the highest observations pointed to windows corresponding with the most pronounced selection signals. Overlapping windows with the highest F_ST_ values were then merged and genomic regions formed by these merged widows were extended on both sides by 25 kb to account for extended linkage and potentially linked genes. The resulting genome regions spanning the strongest diversifying selection signals were analyzed in detail to identify encompassed genes and associated biological processes using the UCSC Genome Browser [6], ENSEMBL database, and PANTHER classification system [7]. The genes overrepresentation tests (in gene ontology categories) were performed according to all annotated horse genes with correction for multiple testing (false discovery rate, FDR). Additionally, the diversifying selection signals were verified and tested for significance using BayeScan software (v2.1) which is based on the multinomial-Dirichlet model [8]. In this software, selection is detected by decomposing F_ST_ coefficients into a population-specific component (beta) shared by all loci, and a locus-specific component (alpha) shared by all populations using logistic regression. Departure from neutrality at a given locus is assumed when the locus-specific component is necessary to explain the observed pattern of diversity (alpha significantly different from 0). A positive value of alpha suggests diversifying selection, whereas negative values suggest balancing or purifying selection. This leads to two alternative models for each locus, including or not the alpha component to model selection. For each locus, a reversible-jump Markov chain Monte Carlo MCMC statistic explores models with and without selection (alpha component being either present or absent, respectively,) and estimates their relative posterior probabilities [9].

The linkage disequilibrium patterns and haplotype block structures at the genome regions with the strongest signs of divergent section identified using initial F_ST_-based approach were analyzed using HaploView 4.2 [10] software examining pairwise linkage disequilibrium (LD) on the distance up to 500 kb and detecting blocks based on a method proposed by Gabriel et al. [11].

To additionally evaluate the genetic structures of the studied populations, we employed principal components analysis (PCA) based on animal genotypes and inter-individual distance matrix calculated as 1 − IBS (identity-by-state) similarity, with IBS defined as the probability that alleles drawn at random from two individuals at the same locus are the same. The obtained IBS distances were then used to create cladograms based on a neighbor-joining (NJ) method [12] and visualized with use of Archaeopteryx Tree software (v0.9928 beta) [13].

To further validate the population structure and the patterns of admixture among populations, Admixture software [14] was used for the K values from 1 to 5. Then, we used Clumpak software [15] to visualize the results. The best K value was identified based on a cross-validation procedure implemented in Admixture software.

A population structure was additionally evaluated by calculating four genomic inbreeding measures: (i) the usual variance-standardized relationship minus 1 (F_GRM_), (ii) method-of-moments F coefficient estimate (similar to F_IS_), (iii) F coefficient based on the correlation between uniting gametes (F_U_) [5], and runs of homozygosity (ROH)-based coefficient (F_ROH_, including ROH segments with lengths above 1 Mb) [16] using Plink v1.90b4 software [4]. The first three coefficients were calculated using Plink --ibc command, while ROH-based coefficient was calculated by identification of ROH covering minimum of 30 SNPs (as in our previous study in cattle [17]) and evaluation of a genome portion covered by ROH as proposed by McQuillan et al. [16]. Effective population sizes (N_e_) were estimated with the use of NeEstimator v2.1 [18] software based on 1000 randomly sampled SNP genotypes to minimize computation time.

## 3. Results

**Figure 2:**
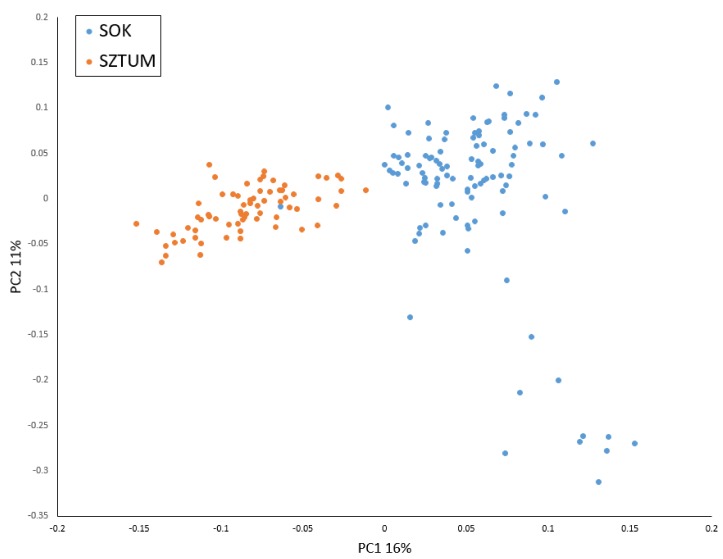
Principal components analysis based on single nucleotide polymorphism SNP genotypes. SOK: Sokólski and SZTUM: Sztumski.

**Figure 3:**
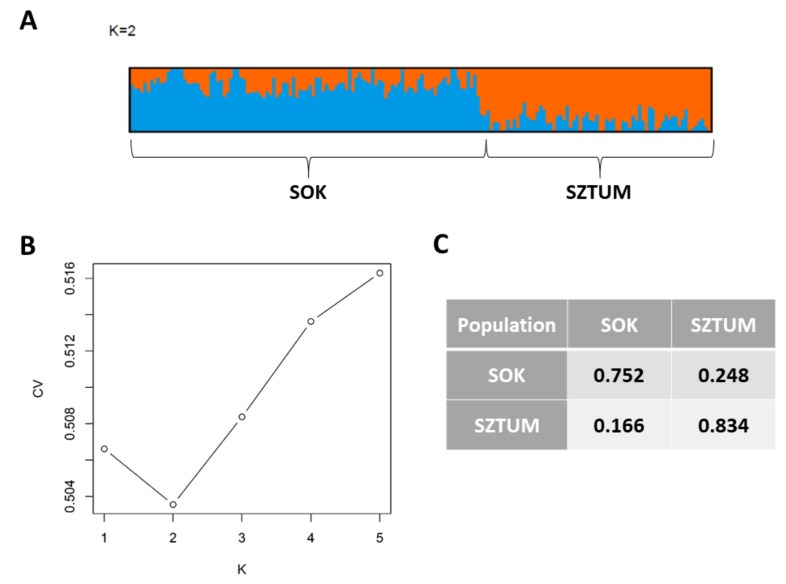
Patterns of admixture among populations obtained using Admixture software for K = 2 (**A**), plot of Admixture cross-validation procedure (**B**), and average membership coefficients (Q) for separate horse types (**C**). SOK: Sokólski, SZTUM: Sztumski, CV: cross validation error, and K: cluster (number of subpopulations).

After initial SNP filtering 52,023 markers were retained for further analysis. SNP panel polymorphism parameters were described in our previous study [3]. In brief, average MAF for the analyzed SNPs was 0.215 in SOK and 0.214 in SZTUM horses. The average observed heterozygosity was also very similar for both horse types and was 0.296 in SOK and 0.297 in SZTUM. The analysis of kinship coefficient obtained from GRM showed overall low relatedness among animals in sampled populations, suggesting no oversampling of related individuals (mean pairwise values of coefficient were close to 0 in both populations). The analysis of the studied individual genotypes with the principal components analysis showed that the two populations formed clear but overlapping clusters with some individuals being genetically close in both populations. The population of the Sokólski horse also included some individuals with genetic profiles visibly differing from other animals within the type (Figure 2). Similar results were obtained when IBS distance matrix was employed to create a cladogram (Figure S1). Most of the individuals of the SOK population clustered together and separately from SZTUM horses with the exception of six individuals clustering together with a subcluster of Sztumski horses. These individuals, however, were not within the overlapping part of clusters in the PCA analysis and were randomly distributed in the center of the SOK cluster. The results obtained with the use of Admixture software showed that the best clustering of the analyzed population divided them into two clusters (K = 2, according to cross-validation procedure), mostly corresponding to the SZTUM and SOK populations (Figure 3). The average membership coefficient of both horse types ranged from 0.524–0.999 in SOK (excluding one individual which was classified with SZTUM with a Q value of 0.267) and 0.539–0.99 in SZTUM. The SOK individual clustering with SZTUM horses (also present in PCA data) is probably a case of misidentification of error in pedigree data. Nevertheless, the results obtained for K = 3 and K = 4 showed some subpopulations within separate types which confirmed previously-observed (using PCA method) subdivision of the SOK population (Figure 2).

The global genetic differentiation of the analyzed populations measured by F_ST_ was low and mean and weighted mean F_ST_ values were 0.012 and 0.014, respectively, suggesting that about 98% of genetic variation within the analyzed horse types can be attributed to inter-individual variation. The genomic inbreeding coefficients were comparable for both populations and depending on the applied measure ranged from 0 for F_GRM_ to about 13% for F_IS_. The average F_ROH_ values for ROH segments with length over 1Mb were also similar for both horse types and showed values of about 11% (Table 1). The estimated effective population size was high for both populations, but higher for Sokólski (228) than for Sztumski (156.4) horses.

**Figure 4:**
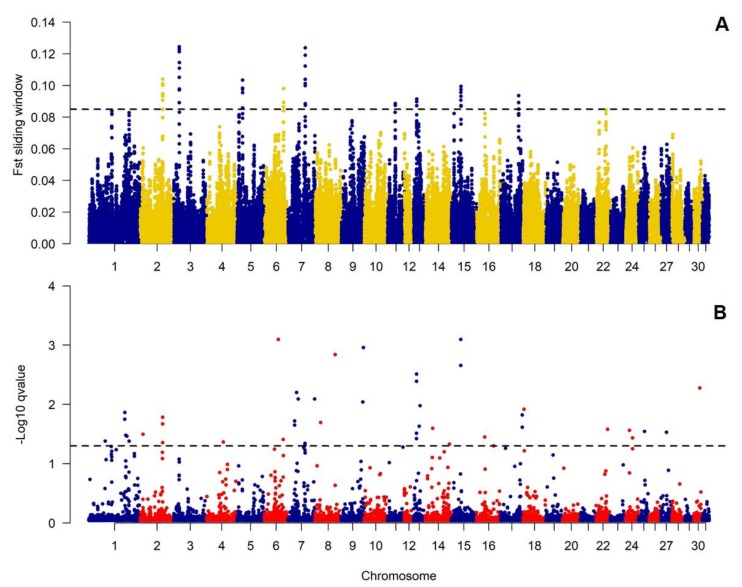
Genome-wide distribution of diversifying selection signatures among Sokólski and Sztumski horses, (**A**) detected with sliding-window approach and (**B**) BayeScan software. In part A, sliding window-averaged F_ST_ values for individual SNPs are plotted against centered genomic positions of the windows. Dashed line shows threshold for the top 0.1% highest F_ST_ values (0.085). In part B, −log10 *p*-values are plotted. Dashed line indicates SNPs with *p*-value < 0.05.

We additionally decided to verify if genetic differences between the studied populations concentrate over specific genomic loci suggested the occurrence of genetic effects of diversifying selection. This analysis showed that the anticipated effects of selection were especially pronounced on ECA2, 3, 5, and 7 (Figure 4, Table S1.). The genomic regions covering all detected selection signals (with sliding-window averaged F_ST_ values higher than 99.9% of all observations) had size from 278 to 826 kb and covered in total 4.3 Mb of the genomic sequence (Table 2). The comparative analysis with BayeScan results showed that the sliding-window-based approach correctly identified most of the signals detected with the Bayesian method (Figure 4). All significant signals (*p*-value < 0.05) had positive Alfa coefficient corresponding to the presence of positive selection (Table S1.). The regions under selection (according to the sliding-window based approach) overlapped with 60 different genes. The genes did not enrich significantly any biological processes or metabolic pathways, however, included inter alia those connected with nucleic acid metabolic process and cellular nitrogen compound metabolic process (Table S2.).

**Figure 5:**
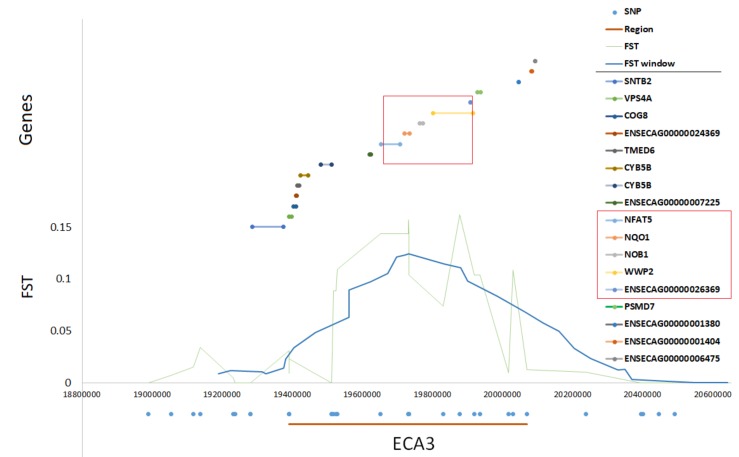
Detailed view of the divergently-selected region on ECA3. Region position, SNP positions, F_ST_ and window averaged F_ST_ values, and gene positions directly at the selected region are plotted.

**Figure 6:**
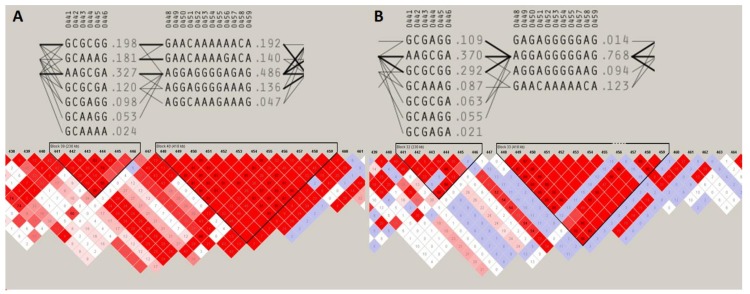
Linkage disequilibrium and haplotype block structure directly at the region divergently selected on ECA3 between Sokólski (**A**) and Sztumski (**B**) horses. Successive SNPs on the chromosome are numbered at the top of the figure. Sequences of haplotypes as well as their frequencies are given below. The thickness of lines connecting haplotypes is dependent on the frequency of common segregation, where thick line indicates stronger linkage. Black triangles mark the haplotype blocks. The colored squares represent the pairwise SNPs linkage disequilibrium as D’ values. D’ = 1.00 in the blank red squares. Numbers inside the squares are r^2^ × 100. Detailed labeling of LD is described in Haploview manual [19].

**Figure 7:**
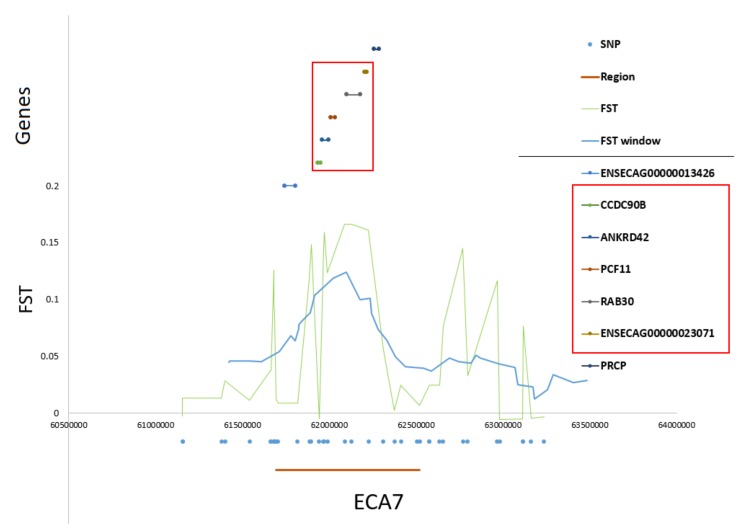
Detailed view of the divergently-selected region on ECA7. Region position, SNP positions, F_ST_ and window-averaged F_ST_ values, and gene positions directly at the selected region are plotted.

**Figure 8:**
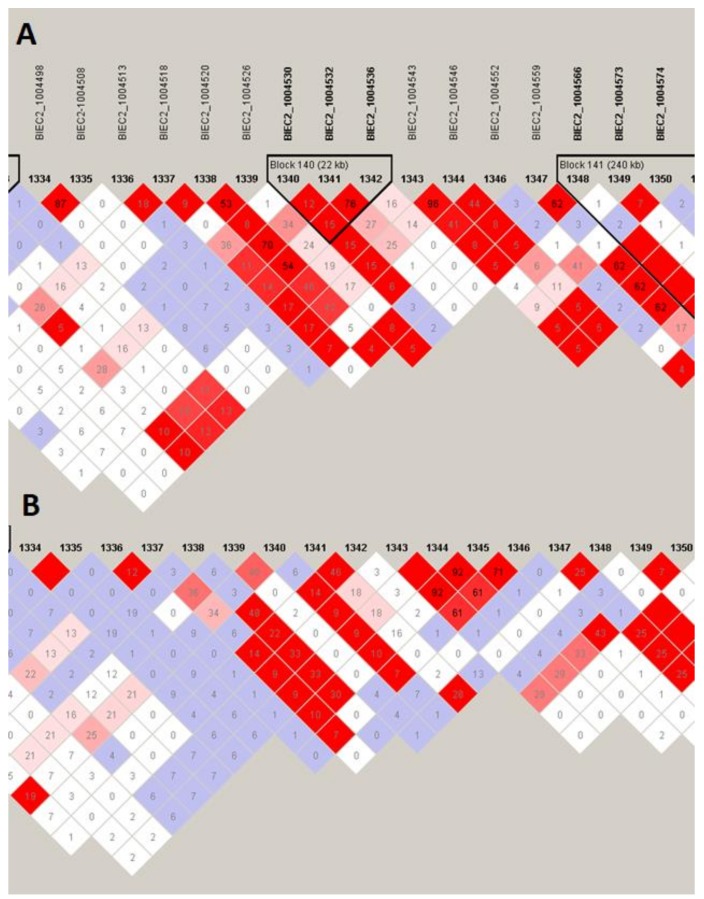
Linkage disequilibrium and haplotype block structure directly at the region divergently selected on ECA7 between Sokólski (**A**) and Sztumski (**B**) horses. Names of SNPs and their successive numbering on chromosome are given at the top of the figure. Black triangles mark the haplotype blocks. The colored squares represent the pairwise SNP linkage disequilibrium. D’ = 1.00 in the blank red squares. Numbers inside the squares are r^2^ × 100. Detailed labeling of LD is described in Haploview manual [19].

The two most pronounced selection signals on ECA 3 and 7 were analyzed in detail to identify potential key genes responsible for the observed differences among the studied populations. We detected 13 well-annotated genes (17 in total) within the region on ECA3. The analysis of F_ST_ values directly at the locus showed that there were five genes including *NFAT5*, *NQO1*, *NOB1*, *WWP2,* and one miRNA gene (*MIR140*) (Figure 5) on the top of the selection signal peak. Additionally, the analysis of linkage disequilibrium and haplotype block structure at the locus showed a high level of LD in both populations (higher in SOK horses) and two haplotype blocks with a presence of individual haplotypes divergently selected between the analyzed horse types (Figure 6). The region on ECA7 encompassed seven genes. The top F_ST_ values overlapped with four well-annotated genes, namely *CCDC90B*, *ANKRD42*, *PCF11,* and *RAB30* (Figure 7). The region was characterized by a moderate level of LD in both populations with only a short, three-SNP haplotype block found in the SOK population (Figure 8). A common haplotype with frequency over 40% was observed within this block, which overlapped with *CCDC90B* and *ANKRD42* genes.

## 4. Discussion

Standard methods of genetic diversity evaluation and management have proved to be effective in conservation of animals’ genetic diversity during several years of animal breeding. Nevertheless, together with the development of molecular and computational sciences, new methods of genetic variability evaluation have become available which may help in more efficient conservation program managing and a more effective breeding decision-making process. These new methods use tools of advanced molecular and population genetics allowing deep analyses of within- and among-population variation and the analysis of population history as well as providing reliable quantitative measures of genetic differentiation even for poorly-characterized populations [20]. Additionally, the currently-applied methods of genomics allow us to study genetic variation not only at global genomic level but also on chromosomal or even regional levels, providing tools to capture the smallest signs of genetic differentiation undetectable with the use of classical or less-advanced molecular methods [21].

The current breeding program of the Sokólski and Sztumski horse assumes separate sections of stud books within cold-blooded horses (since 2008) and aims at clear separation of these populations as separate breeds. This is dictated by the fact that these horses possess their historical, genetic, and phenotypic specificity, resulting from the used breeding material on the basis of which they were produced as well as different environmental conditions which shaped their present character [2]. Thus, in this study we attempted to answer a question asked in recent years by breeding organizations about the exact level of genetic differentiation of Sokólski and Sztumski horses as this is a subject of genetic resource conservation. We also attempted to detect genome regions bearing variants responsible for the observed phenotypical differences between these horses, by the identification of diversifying selection signals. To this end, we used whole-genome genotyping arrays being a standard tool for a variety of genome-wide analyses performed in different horse breeds [20,22].

A previous attempt to characterize genetic differentiation of Sokólski and Sztumski horses with the use of microsatellite markers resulted in similar low estimates of the average genetic distance between those populations (at F_ST_ = 0.03) [23]. This genetic differentiation was also virtually undetectable in our previous study which involved other Polish horse breeds [3]. The approach applied in this study focused specifically on the detection of allele frequency differentiation across the genome between these two populations allowing a clear identification of genetic differences both at global as well as regional genomic levels. The approach used the principal component analysis method as well as IBS distance analysis and showed clear separation of genetic profiles of individuals belonging to separate populations with some possible signs of admixture between them. The results of Admixture software, however, classified individuals according to their type of origin with the best possible segregation of animals into two distinct populations. All these results suggested that despite the low average genetic differentiation of the analyzed populations they are characterized by a detectably-distinct genetic profile which may result from site-specific allele frequency differences and patterns. To verify this assumption, we analyzed locus-by-locus genetic distances and identified genome sites that showed relatively high level of genetic differentiation, presumably including variants being responsible for the observed heritable phenotypic differences between the analyzed populations. These sites were localized on ECA2, 3, 5, and 7, which were previously shown to bear quantitative traits loci (QTLs) e.g., for ECA2, navicular bone morphology; ECA3 and 5, osteochondrosis dissecans and male fertility; and ECA7, gait traits and body height [24]. What is the most important, the specific regions divergently selected on these chromosomes closely co-localized with several QTLs for withers height [24]. Moreover, we observed high levels of LD in both populations and a presence of common haplotypes divergently selected between the analyzed populations within one of the most pronounced selection signals on ECA3, suggesting a regional loss of genetic variation presumably under the influence of the recent artificial selection. The most pronounced selection signal associated with this locus overlapped with *NFAT5*, *NQO1*, *NOB1*, *WWP2,* and *MIR140* genes. While phenotypic differences between Sokólski and Sztumski horses involve mainly body size and constitution, the presumed candidate genes should be connected with e.g., growth, development, and/or metabolism. In this light, the exact role of the genes detected in the region is difficult to explain without building a complex theory unsupported by this study’s results. Similarly, within the another strongly divergently-selected region on ECA7, we detected inter alia *CCDC90B*, *ANKRD42*, *PCF11,* and *RAB30* genes, with functions (Table S2.) which cannot be easily and directly associated with phenotypical traits differing across the analyzed populations. Nevertheless, among the detected genes there were ones responsible for e.g., nucleic acid metabolic process, cellular nitrogen compound metabolic process, MAPK/ERK pathway regulation, and pre-mRNA cleavage; thus the genes can be presumed as important elements of molecular mechanisms leading to manifestation of the observed phenotypical differences. Obviously, it cannot be excluded that the observed differences in allele frequency are caused by genetic drift and random changes in allele frequencies occurring differently in separated populations. However, systematic deviations in allele frequency detected with the window-based and Bayesian approach prompt us to propose artificial selection as a mechanism driving genetic differentiation of the analyzed horse types.

## 5. Conclusions

Altogether, the performed analysis shows that despite a generally low level of genetic differentiation, Sokólski and Sztumski horses are characterized by visible differences in their genetic profiles which may result from both discrete fluctuations of allele frequency across the genome as well as from the differentiation of specific genomic loci being probably associated with the presence of gene variants with a large effect on the differing phenotypical traits of these two horse types. Based on these observations, we can conclude that further maintaining of separate stud books is reasonable and will allow preserving and increasing of the observed genetic differentiation among the studied draft horse populations.

## Figures and Tables

**Table 1:** Genetic diversity parameters for the studied horse populations.

Type	H_o_	H_e_	F_ST_ Mean	F_ST_ Median	N_e_	F_GRM_	F_IS_	F_U_	F_ROH_
Sokólski	0.296	0.295	0.0124	0.0139	228	−0.00083	0.137	0.068	0.110
Sztumski	0.297	0.294			156.4	−0.00368	0.132	0.064	0.107

H_o_: observed heterozygosity, H_e_: expected homozygosity, F_ST_: Wright’s genetic distance, N_e_: effective population size, F_GRM_: G-matrix -derived F coefficient, F_IS_: Wright’s inbreeding coefficient, F_U_: F estimate based on correlation between uniting gametes, and F_ROH_: runs of homozygosity-based F coefficient.

**Table 2:** Genomic regions with the strongest diversifying selection signatures among the analyzed populations according to the sliding-window based approach.

Chromosome	Start	End	Size (bp)
2	80,426,523	80,826,149	399,626
3	19,391,644	20,068,977	677,333
5	19,725,673	20,099,989	374,316
6	67,856,137	68,433,748	577,611
7	61,694,614	62,521,051	826,437
11	27,516,665	27,795,394	278,729
13	10,773,369	11,155,590	382,221
15	34,201,183	34,674,868	473,685
17	63,875,241	64,221,049	345,808

## Supplementary Materials

The following are available online at https://www.mdpi.com/2076-2615/10/3/542/s1: Figure S1: Cladogram created based on identity-by-state distance matrix. Table S1.: Window-averaged FST values across genome between the Sztumski and Sokólski populations and the results of the BayeScan software analysis. Table S2.: genes detected within genome regions covering the strongest-detected diversifying selection signals.
